# Multimodal management of postoperative neuropathic pain and scar-associated nerve entrapment following Mohs surgery

**DOI:** 10.1016/j.jdcr.2025.09.052

**Published:** 2025-10-27

**Authors:** Olga Gomeniouk, Solomiya Grushchak

**Affiliations:** a Rush Medical College, Chicago, Illinois; b VA San Diego Healthcare System Department of Dermatology, San Diego, California; c USA La Jolla Cosmetic Laser Clinic, San Diego, California; d USA Chicago Cosmetic Surgery and Dermatology, Chicago, Illinois

**Keywords:** CO2 laser, Mohs surgery, nerve entrapment, neuropathic pain, scar

## Introduction

Nerve entrapment within scar tissue after surgery can result in pain and functional impairments, posing a complex therapeutic challenge. Scar formation can compress or entrap nerves, causing persistent numbness, tingling, or pain. Among the available modalities, fractional carbon dioxide (CO_2_) laser therapy has emerged as a promising modality due to its dual capacity for scar remodeling and enhanced drug delivery.^1^

Fractional CO_2_ lasers create microthermal treatment zones, controlled vertical channels that facilitate the precise delivery of topically applied agents. This method, termed laser-assisted drug delivery, augments the penetration and bioavailability of therapeutics such as corticosteroids, used to reduce inflammation and fibrosis.^2^ Compared to other ablative lasers such as erbium-doped yttrium aluminum garnet, CO_2_ lasers penetrate deeper and have a coagulative effect, making them particularly suited for delivering high molecular weight molecules into the dermis, promoting sustained drug diffusion and improved outcomes.^3^

The tunable nature of CO_2_ lasers allows for customizable depth and density, enabling targeted scar modulation while preserving surrounding tissue integrity.^4^ This capability is especially advantageous for addressing nerve entrapment, as CO_2_ lasers can release trapped nerve fibers, potentially alleviating associated neuropathic pain.^5^ Enhanced permeability also supports the use of lower topical dosages, reducing systemic and cutaneous side effects, or alternatively, allows for the targeted accumulation of the drug to optimize local effects.^6^

This report presents the successful use of fractional CO_2_ laser-assisted corticosteroid delivery in treating a patient with nerve entrapment and symptomatic scar tissue following Mohs surgery. This approach led to significant pain relief and improved scar texture, demonstrating its utility in complex surgical scars with associated neuropathic pain.

## Case report

**Fig 1:**
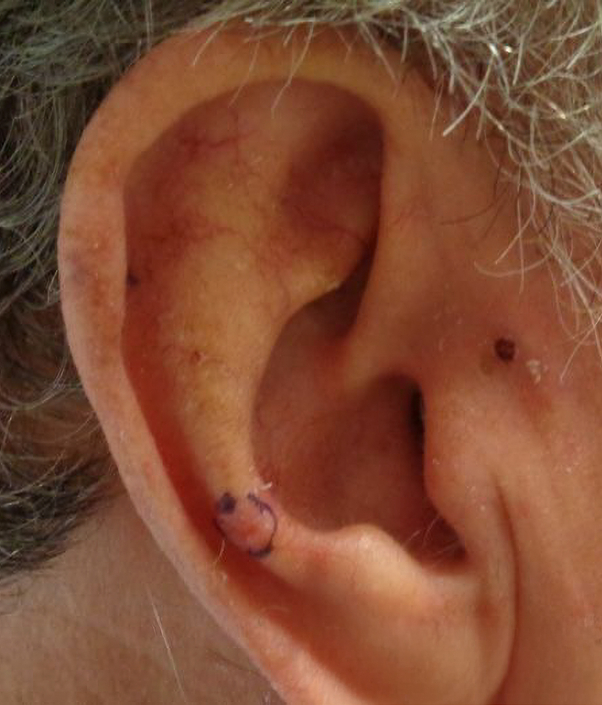
Original biopsy site.

**Fig 2:**
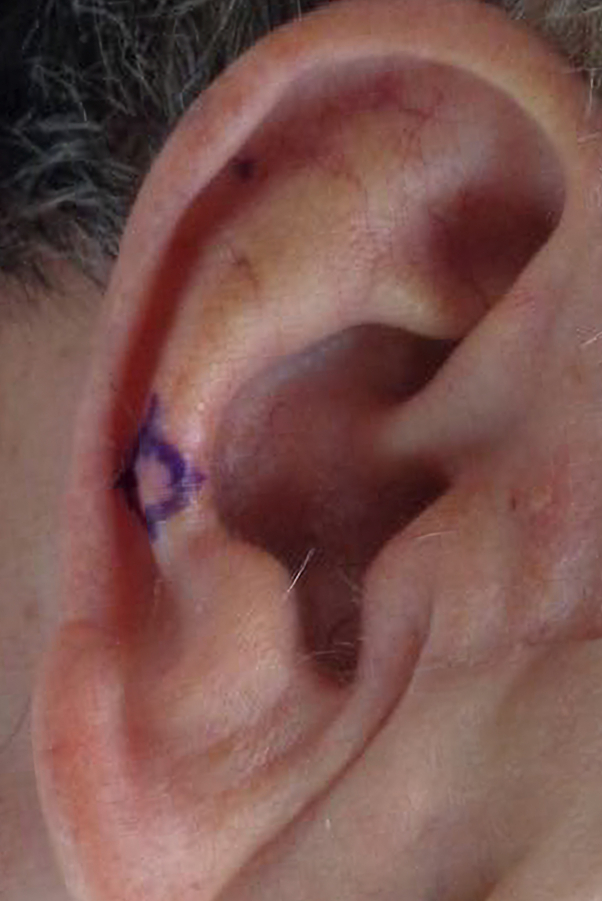
Preoperative site.

A 75-year-old male with chronic kidney disease, masseteric hypertrophy, temporomandibular joint pain, and history of gastric bypass surgery presented for surgical resection of a basal cell carcinoma of the right antihelix, measuring 0.9 cm × 0.8 cm. The tumor required 4 stages of Mohs micrographic surgery to achieve clear margins. A wedge resection was performed, excising a full-thickness triangular section from the auricle, meticulously planned to preserve the ear's natural contour and as much healthy tissue as possible, resulting in a final defect of 2.2 cm × 1.7 cm. Reconstruction was performed using a bilateral wedge advancement flap with circumferential undermining to facilitate flap mobilization. The flap was elevated and secured in place after the redundant tissue was excised. Closure was performed using 5-0 vicryl subcutaneous sutures and 5-0 fast gut superficial sutures. The procedure was well tolerated with no immediate complications, and the final flap measured 2.2 cm × 0.2 cm. The patient completed a 2-week course of doxycycline 100 mg twice daily for infection prevention (Figs 1 and 2).

**Fig 3:**
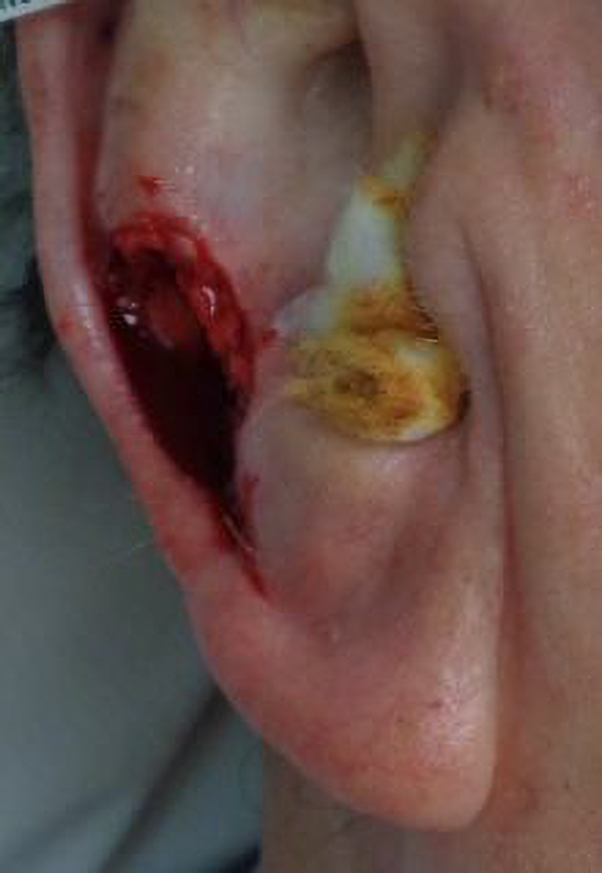
Defect.

Six weeks after surgery, the patient developed persistent auricular pain and worsening of his original temporomandibular joint (TMJ) pain, accompanied by tissue edema in the ipsilateral jowl (Fig 3). Examination revealed a well-healed surgical site without erythema or tenderness. Clinical assessment suggested perichondritis and possible postsurgical nerve entrapment, compounded by masseteric hypertrophy. Nonsteroidal anti-inflammatory agents were contraindicated due to chronic kidney disease and gastric bypass surgery; thus, initial management included gabapentin 100 mg TID PRN for neuropathic pain and 1 cc kenalog injection 10 mg/ml to address inflammation. TMJ symptoms were managed by his neurology team with ongoing botulinum toxin injections and lymphatic massage.

**Fig 4:**
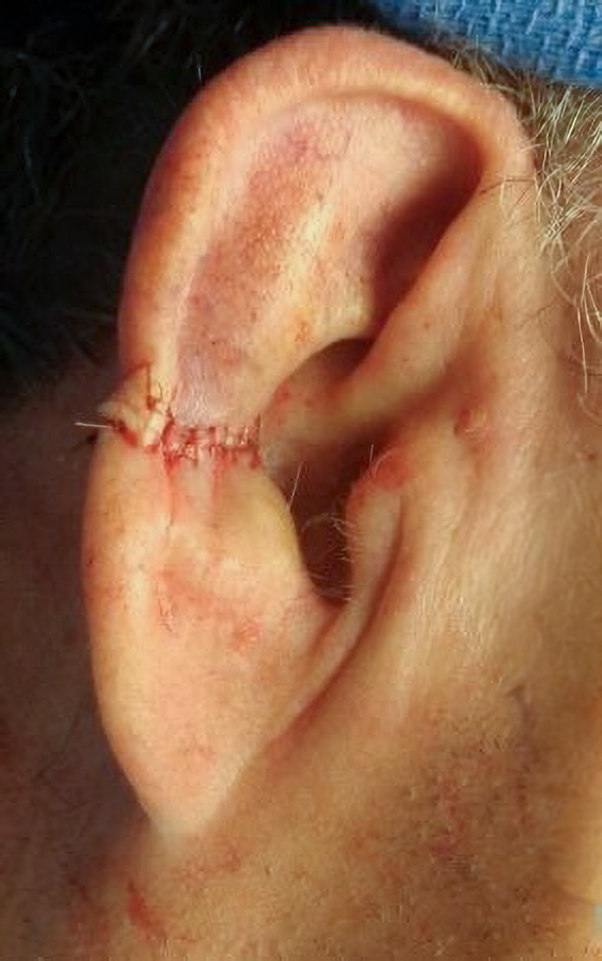
Postoperative site.

**Fig 5:**
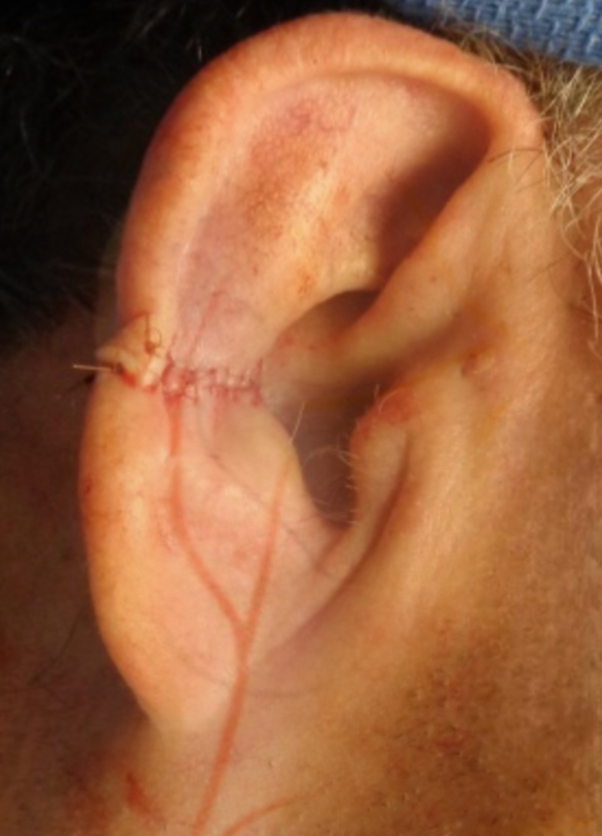
Postoperative site with overlying GAN.

**Fig 6:**
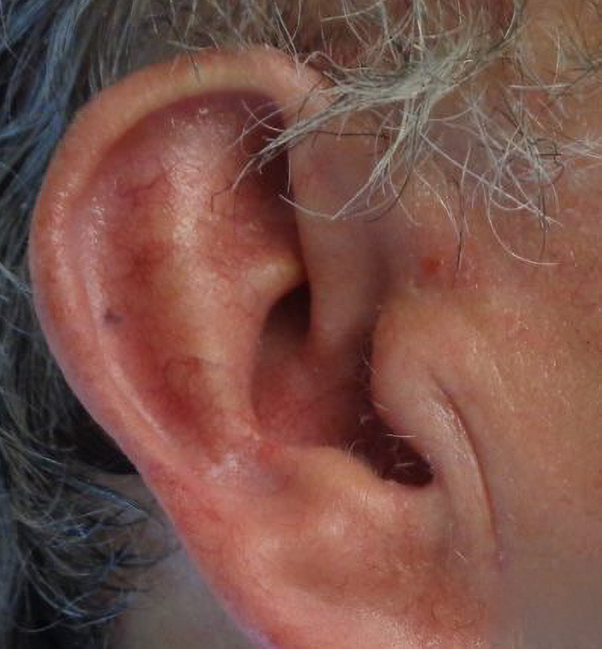
Nine month postoperative site.

To address potential nerve entrapment, a fractional CO2 laser was initiated. One pass of Deep FX was performed at 40 mJ, achieving a depth of 1.2 mm with a density of 5%, followed by topical Kenaolog 10 mg/ml. One week later, the patient reported relief of his auricular pain, noting improvement from 8/10 to 4/10 on the Likert pain scale. He received subsequent treatment with the same 40 mJ and 5% density CO2 laser 4 weeks after initial treatment (Figs 4 and 5). At 9-month follow-up, patient pain has completely resolved (Fig 6).

## Discussion

This case highlights the management of postoperative complications of Mohs micrographic surgery and subsequent reconstruction, such as nerve entrapment resulting in neuropathic pain. The surgery successfully removed the malignancy and achieved advanced closure of the defect that had infiltrated the cartilage. However, the patient’s postoperative course demonstrated the complexity of management in a patient with multiple comorbidities, necessitating an alternative approach to pain management.

The development of perichondritis and worsening TMJ pain, compounded by masseteric hypertrophy and possible nerve entrapment, reflects the multifactorial nature of surgeries involving the head and neck. Neuropathic pain syndromes are well-documented postoperatively, with cutaneous nerve entrapments causing post-surgical pain in up to 30% of cases.^7^ In this patient, nonsteroidal anti-inflammatory agent contraindications necessitated a multimodal pain management approach. This patient had a history of TMJ pain, exacerbated post-surgically, likely related to the nerves affected throughout the procedure. Specifically, helix surgery, such as wedge resection, could exacerbate or trigger TMJ pain due to the region’s complex sensory innervation. The mid-helix typically receives sensory input from the greater auricular nerve , the auriculotemporal nerve, and sometimes the lesser occipital nerve. The GAN, from the cervical plexus, provides sensation to the ear and the angle of the mandible, while the auriculotemporal nerve innervates both the ear and the TMJ, and the lesser occipital nerve, though primarily serving the scalp and posterior auricle, may contribute to helix sensation.^8^

During helix surgery, irritation or compression of these nerves may produce referred pain via sensory convergence in the brainstem. The brain may misattribute the source of pain to the TMJ due to its shared sensory innervation with the auriculotemporal nerve, thereby exacerbating or mimicking TMJ-related discomfort.^9^^,^^10^

Although corticosteroid injections, gabapentin, and botulinum toxin provided partial relief, persistent symptoms suggested the potential for an underlying nerve entrapment with associated perichondritis. This prompted use of CO2 ablative fractional laser therapy, combined with postlaser triamcinolone application to target both inflammatory and neuropathic components. CO2 lasers have demonstrated potential in treating postsurgical neuralgia by stimulating tissue remodeling, reducing fibrosis, and potentially alleviating nerve compression.^1^^,^^4^^,^^5^

In conclusion, this case underscores the importance of a multimodal approach to managing postsurgical complications, utilizing advanced modalities such as fractional laser therapy with corticosteroid delivery, which may provide significant relief and enhance outcomes and quality of life in select patients.

## Conflicts of interest

None disclosed.
